# Structural Interactions within the Default Mode Network Identified by Bayesian Network Analysis in Alzheimer’s Disease

**DOI:** 10.1371/journal.pone.0074070

**Published:** 2013-08-28

**Authors:** Yan Wang, Kewei Chen, Li Yao, Zhen Jin, Xiaojuan Guo, the Alzheimer's Disease Neuroimaging Initiative

**Affiliations:** 1 College of Information Science and Technology, Beijing Normal University, Beijing, China; 2 Banner Alzheimer’s Institute and Banner Good Samaritan PET Center, Phoenix, Arizona, United States of America; 3 State Key Laboratory of Cognitive Neuroscience and Learning, Beijing Normal University, Beijing, China; 4 Laboratory of Magnetic Resonance Imaging, Beijing 306 Hospital, Beijing, China; University G. D'Annunzio, Italy

## Abstract

Alzheimer’s disease (AD) is a well-known neurodegenerative disease that is associated with dramatic morphological abnormalities. The default mode network (DMN) is one of the most frequently studied resting-state networks. However, less is known about specific structural dependency or interactions among brain regions within the DMN in AD. In this study, we performed a Bayesian network (BN) analysis based on regional grey matter volumes to identify differences in structural interactions among core DMN regions in structural MRI data from 80 AD patients and 101 normal controls (NC). Compared to NC, the structural interactions between the medial prefrontal cortex (mPFC) and other brain regions, including the left inferior parietal cortex (IPC), the left inferior temporal cortex (ITC) and the right hippocampus (HP), were significantly reduced in the AD group. In addition, the AD group showed prominent increases in structural interactions from the left ITC to the left HP, the left HP to the right ITC, the right HP to the right ITC, and the right IPC to the posterior cingulate cortex (PCC). The BN models significantly distinguished AD patients from NC with 87.12% specificity and 81.25% sensitivity. We then used the derived BN models to examine the replicability and stability of AD-associated BN models in an independent dataset and the results indicated discriminability with 83.64% specificity and 80.49% sensitivity. The results revealed that the BN analysis was effective for characterising regional structure interactions and the AD-related BN models could be considered as valid and predictive structural brain biomarker models for AD. Therefore, our study can assist in further understanding the pathological mechanism of AD, based on the view of the structural network, and may provide new insights into classification and clinical application in the study of AD in the future.

## Introduction

Alzheimer’s disease (AD) is a well-known neurodegenerative disease that is characterised by abnormal brain anatomy with clinical manifestations of memory loss and cognitive impairment. As a non-invasive technique, structural magnetic resonance imaging (MRI) makes it possible to measure and investigate morphological alterations in the human brain. Using structural MRI, the previous studies have demonstrated that brain volume reductions were a general feature in aging [1], [2] and not only AD specific. However, AD could be taken as the accelerated aging progress, for example, the annualized rate of hippocampal volume loss was higher in AD patients than that in normal controls (NC) [3]. Therefore, AD would lead to extensive volume decreases or severer atrophy in some brain regions compared with NC. Specially, a large number of structural MRI studies have shown that AD patients revealed significant reductions in grey matter, mainly in the medial temporal lobe, the posterior cingulate gyrus, and the parietal and frontal lobes [4], [5], [6], [7], and white matter decreases in the corpus callosum (CC), the inferior longitudinal fasciculus, and the parahippocampal, inferior parietal and middle frontal regions [8], [9], [10], [11]. Most of these studies utilised univariate statistical approaches and focused on localising the affected brain regions. In contrast to univariate methods, multivariate approaches effectively characterised the interrelationships among different brain regions and contributed to the understanding of structural covariance patterns of morphological abnormalities caused by normal aging and dementia [12], [13], [14].

Recently, several studies have used multivariate approaches to identify AD-related covariance patterns [15], [16], [17]. Using a scaled subprofile model (SSM), Alexander et al. investigated the grey matter network in individuals at risk for AD [15]. Multivariate network analyses based on a principal component analysis (PCA) were also used to explore white matter tract integrity in AD [16]. Our previous study utilised joint independent component analysis (jICA) and identified three grey-white matter source networks, mainly in the frontal/parietal/temporal-superior longitudinal fasciculus/corpus callosum regions, that had significant volume reductions in AD patients compared to NC [17]. The approaches were data-driven and voxel-based. Additionally, using a small world approach, i.e., a region of interest (ROI)-based multivariate, He et al. found both decreases and increases in cortical thickness intercorrelations in the parietal and temporal cortices, implying aberrant changes in cortical morphometry in AD patients [18], [19]. Although these multivariate methods provided an effective tool for constructing structural covariance patterns in the study of AD, to date, less is known about specific structural probabilistic dependency or the interactions among spatially distributed brain regions.

In recent years, Bayesian network (BN) analysis without a prior model has been successfully introduced to functional MRI (fMRI) and structural MRI studies [20], [21], [22], [23]. In the context of neuroimaging studies, BN is a ROI-based multivariate technique. Chen et al. performed BN analysis on structural MRI data from individuals with mild cognitive impairment (MCI) and revealed complex, nonlinear multivariate interactions among grey matter volume changes in the left hippocampus and the right thalamus [21]. Furthermore, Chen et al. applied dynamic BN modelling to represent volume-change dependencies among different brain regions over time in a longitudinal study of normal aging and MCI [20]. These studies focused on the brain regions that were significantly AD-related. It would be interesting to explore inter-regional associations among brain regions that are not only affected by AD (or MCI), but are also known for their great importance in general; for example, the core ROI of the default mode network (DMN).

Indeed, the DMN is one of the most frequently studied resting-state networks. Using both fMRI and diffusion tensor imaging (DTI) technology, some researchers now believe that functional connectivity in the resting-state reflects structural connectivity in the DMN, such as the cingulum bundle connecting posterior cingulate cortex (PCC) and the medial prefrontal cortex (mPFC) and the descending cingulum bundles connecting PCC and medial temporal lobe [24], [25]. Although fMRI studies suggested altered DMN connectivity in patients with AD compared to NC [23], [26], it was commonly assumed that the altered functional connectivity was associated with abnormal structural connectivity in the DMN [27], [28] and little was understood regarding structural inter-regional interactions within the DMN. Most of the previous studies on structural covariance patterns in the brain defined the regions involved in the functional network as seeding regions or ROI in order to explore the underlying structural networks [12], [27], [29], [30]. Such studies provided new insights into structural interactions within the DMN in AD.

In this study, we performed a BN analysis by treating regional grey matter volumes as continuous variables to investigate structural inter-regional relationships within the DMN in AD patients in contrast to NC. Eight ROIs in the DMN were defined as nodes of the BN. Then, a permutation test was used to detect differences in the BN models between the AD and NC groups. Furthermore, we evaluated the replicability and stability of our AD-associated BN model in an independent dataset acquired using a different scanner.

## Materials and Methods

### Ethics statement

The Alzheimer's Disease Neuroimaging Initiative (ADNI) study was approved by Institutional Review Board (IRB) of each participating site including Banner Alzheimer’s Institute, and was conducted in accordance with Federal Regulations, Internal Conference on Harmonization (ICH) and Good Clinical Practices (GCP). Data collection for Open Access Series of Imaging Studies (OASIS) was approved by the Washington University Alzheimer Disease Research Center (ADRC) and all the subjects participated in the studies according to the guidelines of the Washington University Human Studies Committee. Written informed consent was obtained from all participants or legally authorized representatives prior to scanning, according to the local IRB rules and local laws.

### Subjects and MRI acquisition

Two datasets, one from the ADNI (http://www.loni.ucla.edu/ADNI) and one from OASIS database (http://www.oasis-brains.org), were used in this study. In order to further guarantee the quality of all the data, we checked every structural image and found that all the images in this study were available.

#### ADNI data

As a $60 million, 5-year project, the ADNI was launched in 2003 by the National Institute on Aging (NIA), the National Institute of Biomedical Imaging and Bioengineering (NIBIB), the Food and Drug Administration (FDA), private pharmaceutical companies and non-profit organizations. The primary goal of ADNI has been to test whether serial MRI, (positron emission tomography) PET, other biological markers, and clinical and neuropsychological assessment can be combined to measure the progression of MCI and early AD. The Principle Investigator of this initiative is Michael W. Weiner MD, VA Medical Center and University of California-San Francisco. ADNI is the result of efforts of many co-investigators from a broad range of academic institutions and private corporations, and subjects have been recruited from over 50 sites across the USA and Canada.

According to ADNI protocols, the severity of cognitive impairment was assessed using Mini-Mental State Examination (MMSE) [31] and Clinical Dementia Rating (CDR) scores [32]. Individuals assigned to the probable AD group met the National Institute of Neurological and Communicative Disorders and Stroke/Alzheimer’s Disease and Related Disorders Association (NINCDS/ADRDA) criteria [33]. At the time this study was initiated, 80 AD patients (39 females and 41 males, mean age: 75.44±6.51 years, range: 60−90; mean MMSE: 23.59±1.92, range: 20−26; CDR: 0.5 or 1) and 101 NC (45 females and 56 males, mean age: 75.93±4.35 years, range: 60−90; mean MMSE: 29.10±1.01, range: 25−30; CDR: 0) were included. The AD group did not significantly differ from the NC group in sex ratio (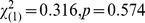) or age (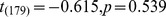) but had significantly lower MMSE scores (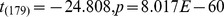). All data were collected at baseline or screening and were acquired on 1.5 T MRI scanners. T1-weighted sagittally oriented 3D anatomical imaging data for each subject was collected using MPRAGE sequence with 1.25×1.25 mm in-plane spatial resolution and 1.2 mm thickness (8° flip angle); the other parameters differed at each scanning site. For each subject, we selected the best quality image which underwent complete pre-processing including B1correction, non-uniformity correction using N3 histogram peak sharpening algorithm and scaling [34].

#### OASIS data

The second dataset included 41 AD patients (75.58±6.94 years [range: 61−92], 17 males and 24 females) with mean MMSE scores of 21.63±3.62 (range: 15−26) and 55 NC (74.16±7.66 years [range: 60−90], 22 males and 33 females) with mean MMSE scores of 29.15 ±1.25 (range: 25−30). All the subjects underwent the full clinical assessment of Washington University Alzheimer Disease Research Center (ADRC) including MMSE and CDR scores. The AD group did not significantly differ from the NC group in sex ratio (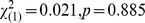) or age (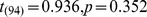) but had significantly lower MMSE scores (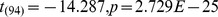). The structural MRI scanning performed on a 1.5 T MRI scanner. For each subject, three to four T1-weighted MPRAGE images were collected (TR = 9.7 ms, TE = 4.0 ms, TI = 20 ms, flip angle  =  10^0^, field of view  =  256 mm × 256 mm, voxel size  =  1 mm × 1 mm, slices  =  128 and thickness  =  1.25 mm, sagittal). In this study, the T1 image was an average image (1 mm × 1 mm × 1 mm) that was a motion-corrected coregistered average of all available data.

For the ADNI and OASIS datasets, there were no significant differences in sex ratio (AD group: 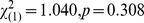; NC group: 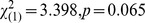) and age (AD group: 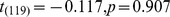; NC group: 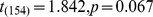). AD group of ADNI dataset significantly differed from that of OASIS dataset in the MMSE scores (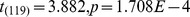), but NC group did not (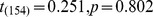).

### Image preprocessing

The spatial preprocessing of the structural MRI data was performed using the VBM8 Toolbox (http://dbm.neuro.uni-jena.de/vbm8) in SPM8 (http://www.fil.ion.ucl.ac.uk/spm). The VBM8 procedure involved two main steps: segmentation and normalization. For each subject, every structural image was segmented into a rigid-body aligned grey matter, white matter and cerebrospinal fluid (CSF) using adaptive maximum posterior and partial volume estimation [35], [36]. Two denoising methods, spatially adaptive non-local means denoising filter [37] and classical Markov Random Field approach were applied to improve the segmentation. The grey matter image was normalized by a diffeomorphic anatomical registration using exponential Lie algebra (DARTEL) protocol [38], in which template creation and image registration are performed iteratively. DARTEL utilises a single constant velocity field to generate diffeomorphic and invertible deformations. At each iteration, the individual brain tissue maps were registered to a newly created template, and, finally, the grey matter tissue maps were normalized to the Montreal Neurological Institute (MNI) space. Afterwards, the registered grey matter maps were multiplied by Jacobian determinants with only non-linear warping to exclude individual differences in total intracranial volume. Lastly, the grey matter maps for all the subjects were smoothed using an 8 mm full width at half maximum (FWHM) Gaussian kernel.

For the OASIS data, spatial preprocessing was performed following the same procedures used for the ADNI data.

### ROIs definition

We named eight ROIs in the DMN mainly according to the previous studies [39], [40]. Each ROI mask was generated using WFU_PickAtlas software (http://www.ansir.wfubmc.edu) [41], [42]. Table 1 shows the specific details of eight ROIs. Every ROI mask covered the entire area of the corresponding anatomical region defined by the AAL altas. We respectively positioned the ROI masks to cover the entire grey matter map for each subject. The average grey matter volume of each ROI was calculated by thresholding all the voxels in the smoothed grey matter images within the ROI at a level of 0.15 for each individual. For the ADNI data, the average grey matter volumes of the eight ROIs, as the nodes of the BN, were used as continuous variables that were inputted into the BN model to investigate structural interactions within the DMN in AD patients and NC. For the OASIS data, the ROIs definition was implemented following the methods described above.

**Table 1:** Eight ROIs in the DMN.

Brain regions	Abbreviations	AAL labels
Posterior cingulate cortex	PCC	cingulum_post_L/R + precuneus_L/R
Medial prefrontal cortex	mPFC	frontal_sup_medial_L/R
Left hippocampus	lHP	hippocampus_L+parahippocampus_L
Right hippocampus	rHP	hippocampus_R+parahippocampus_R
Left inferior parietal cortex	lIPC	parietal_inf_L
Right inferior parietal cortex	rIPC	parietal_inf_R
Left inferior temporal cortex	lITC	temporal_inf_L
Right inferior temporal cortex	rITC	temporal_inf_R

### Bayesian network analysis

A BN model, consisting of nodes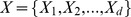 and directed arcs, is a directed acyclic graph (DAG) that can be used to describe conditional dependence among nodes [21], [23], [43]. Below, we provide a brief introduction to BN in the context of the current study.

For the *j^th^* of *d* = 8 ROIs, 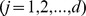, 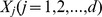 is a set of average grey matter volumes of ROI 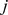. Given the variable 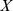, we applied the search-and-score approach to generate the graph structure and maximum likelihood estimation to obtain a set of parameters [22]. Bayesian Information Criterion (BIC) was used as the model evaluation criterion when searching for the best model. 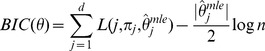



Here,*d* and *n* are the number of nodes and the number of the sample, respectively; 
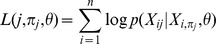
 is the log-likelihood of node *j* with parent node-set 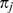, indicating the degree of fitness; 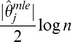 is the penalty of model complexity; 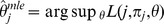 is the maximum likelihood estimate of the parameter of node *j*. The procedure was implemented based on the Bayesian Net Toolbox (www.cs.ubc.ca/~murphyk/Software/BNT/bnt.html).

For each node *j*, the expression 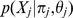 represents the conditional probability density of the node given its parent node-set 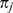. The joint probability density for all the ROIs is:
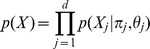


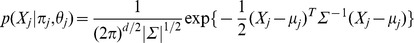
where 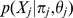 is the conditional probability density of node *j*; 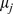 and 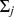 denote the conditional mean and variance of 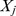 respectively.

Each node in the BN model can be considered as a linear function of its parent nodes with an additional error term. A permutation test [44] was employed to examine the difference in each of the linear coefficients between the AD and NC groups for each node. In our study, permutation was performed 5000 times on the distributions of all subjects, and we assessed the difference in terms of the type-I error probability of AD> NC or NC>AD.

BN models as a classification tool: generalizability evaluation using an independent dataset

We first utilised the ADNI data to construct BN models for the AD and NC groups. We then used the OASIS data to evaluate the distinguishing power of the models in the classification of AD and NC. By comparing the joint probability density between the two BN models, we could predict the group membership of a given subject. Finally, classification accuracy was assessed using receiver operating curve (ROC) analysis. We defined accuracy as N_Y_/(N_Y_+N_N_), in which N_Y_ was the number of individuals that were correctly identified and N_N_ was the number of individuals that were not correctly identified.

## Results

### Structural interactions within the DMN

**Figure 1:**
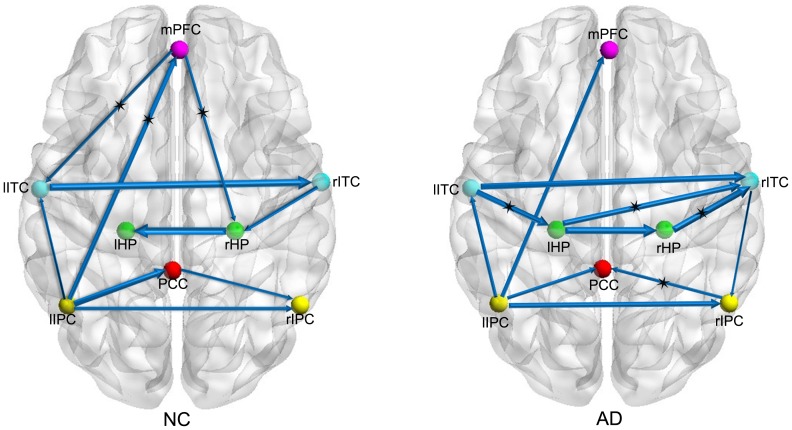
Bayesian network models of DMN based on grey matter volume variations in NC (left panel) and AD (right panel). The arrows represent dependency among brain regions and the thickness of the arrows represents the strength of the connections. The connections with asterisks are those that were significantly stronger for NC/AD than for AD/NC.

Figure 1 shows the BN models of the AD and NC groups, and each connection direction and weight coefficient are given in Table 2. Connections, including lITC_rITC, lIPC_rIPC, lIPC_PCC, lIPC_lITC and lIPC_mPFC, were present in both the AD and NC groups. Although the connections between rHP and lHP, rITC and rHP, rIPC and PCC were observed in both groups, the directions of the connections were opposed to each other. The connections from mPFC to lITC and from mPFC to rHP were observed only in the NC group, and the connections from lITC to lHP, rITC to rIPC, and lHP to rITC were only observed in the AD group.

**Table 2:** List of connections and corresponding weight coefficients in the Bayesian network models of the NC and AD groups.

		Weight coefficients	
Connections		NC	AD
**I**	lITC_rITC	0.8118	0.7026
	lIPC_rIPC	0.4035	0.5992
	lIPC_PCC	0.6841	0.4065
	lIPC_ lITC	0.3021	0.3930
	lIPC_mPFC	0.5990	0.4888
**II**	mPFC_lITC	0.3506	
	mPFC_rHP	0.3199	
**III**	lITC_lHP		0.6596
	rITC_rIPC		0.2626
	lHP_rITC		−0.6363
**IV**	rHP_lHP	0.8421	
	lHP_rHP		0.8023
	rITC_rHP	0.4000	
	rHP_rITC		0.7691
	PCC_rIPC	0.3397	
	rIPC_PCC		0.3723

Note: Part I of this table lists connections in both the AD and NC groups. Parts II and III list the connections present in only the NC or AD, respectively. Part IV lists connections with opposing direction for the two groups.

### Between-group interactions differences

The permutation test-based type-I errors for the between-group differences are listed in Table 3. At a significance level of_, the connections from lIPC to mPFC, mPFC to lITC, and mPFC to rHP in the NC group were stronger than in the AD group. Additionally, compared to the NC group, the connections from lITC to lHP, lHP to rITC, rHP to rITC, and rIPC to PCC were stronger in the AD group.

**Table 3:** Type-I error probabilities of the between-group connection differences.

NC>AD		AD>NC	
Connections	Probability	Connections	Probability
lITC_rITC	0.4044	lITC_rITC	0.5956
lIPC_rIPC	0.7514	lIPC_rIPC	0.2486
lIPC_PCC	0.3330	lIPC_PCC	0.6670
lIPC_lITC	0.7002	lIPC_lITC	0.2998
**lIPC_mPFC**	**0.0000**	lIPC_mPFC	1.0000
**mPFC_lITC**	**0.0282**	**lITC_lHP**	**0.0380**
**mPFC_rHP**	**0.0476**	rITC_rIPC	0.1586
rHP_lHP	0.1638	**lHP_rITC**	0.0038
rITC_rHP	0.1580	lHP_rHP	0.1454
PCC_rIPC	0.1920	**rHP_rITC**	**0.0046**
		**rIPC_PCC**	**0.0352**

Note: The left column, “NC>AD”, shows the probabilities of type-I error in the hypothesis that connections in NC group are greater than in the AD group. The right column, “AD>NC”, displays the opposite situation. The probabilities in bold indicate significantly greater connections (*p*<0.05).

### Classification accuracy as assessed by the second dataset

The classification results for the ADNI and OASIS data are summarised as follows. The classification accuracies of the two datasets were similar, 84.53% for ADNI and 82.29% for OASIS. The corresponding joint probability density scores distinguished the AD patients from NC with 87.12% and 83.64% specificity, 81.25% and 80.49% sensitivity for the ADNI and OASIS data, respectively.

## Discussion

In this study, we constructed two BN models to investigate the structural interactions of grey matter among the core regions of the DMN in AD and NC. We employed a permutation test to detect differences in BN connections between the two groups. The AD patients showed significant reductions in inter-region dependency in the connections from lIPC to mPFC, mPFC to lITC, and mPFC to rHP and increases in the connections from lITC to lHP, lHP to rITC, rHP to rITC, and rIPC to PCC. Moreover, the application of the constructed BN based on the ADNI data predicted AD and NC in a second dataset with high accuracy, sensitivity and specificity.

 The connections between the bilateral brain regions located in the left and right hemispheres were strong in both the AD and NC groups. And the connection patterns between bilateral regions including connections from lITC to rITC and lIPC to rIPC were consistent with the study of functional connectivity in the DMN conducted by Wu et al.[23]. In a previous study, Zheng et al. proposed that functional activation was distributed in both hemispheres and that the left hemisphere might influence the right hemisphere via the anatomical connections of the CC [22]. In addition, Mechelli et al. demonstrated that the grey matter density of a brain region could be used to predict the density of the same region in the contralateral hemisphere [45]. The findings from these two studies are in agreement with the significant bilateral connections shown in the current study. In our study, an interaction between the PCC and the lIPC was observed in both groups but there was no between-group difference. In a previous study, Pagani et al. found that the PCC covaried with the left lateral parietal lobe by using single photon emission computed tomography (SPECT) [46]. Moreover, a connection between the mPFC and the lIPC existed in both groups, with evidence that the inferior parietal lobule was connected to the prefrontal cortices [47].

Significantly decreased interactions among the mPFC and the lIPC, lITC, and rHP were found in the AD group. These decreased connections might have resulted from the lack of brain plasticity which referred to the brain’s lifelong ability for physical and functional changes during maturation, learning and environmental influence [48]. The frontal regions are related to memory and executive function, and the mPFC is an important hub in whole brain connectivity [49], [50]. In this study, the mPFC had interactions with different brain regions in NC group revealing that the mPFC was an important hub to exchange information with other DMN core regions. Compared to NC, we found that the number of connections between the mPFC and other brain regions was reduced in AD which might arise from the atrophy of grey matter affected by AD. The mPFC might be vulnerable in AD due to its anatomical connections with regions firstly affected by this disease. Specifically, the HP, playing an important role in memory, atrophied at the early stage and had connection with mPFC, but this connection was lost in AD [51], which was consistent with the current study. Using DTI technique, Lo et al. found that the decreased nodal efficiency in AD was mainly located in the frontal regions, including the medial part of the superior frontal gyrus, the dorsolateral part of the superior frontal gyrus, and the middle frontal gyrus [49]. Our results agreed with the research conducted by Lo et al. in this regard. We observed that the interaction between the mPFC and PCC was lost in two groups, which might arise from the injured cingulate bundle caused by aging [52].

Compared to the NC group, there were some significantly increased interactions in the AD group, including lITC_lHP, lHP_rITC, rHP_rITC and rIPC_PCC. During MCI and early AD, regional grey matter atrophy was mainly located in the hippocampus and then extended to other brain regions as the disease progressed [4], [21]. In addition, the strength of the interaction from lIPC to lITC in AD was increased, although the between-group difference was not prominent. In the BN models, we observed a probabilistic dependency of lIPC_mPFC_lITC in the NC group but not in the AD group. Disruption of this probabilistic dependency in the AD group might be due to the abnormality in the mPFC; therefore, the lITC might predominantly rely on the lIPC to some extent, which could lead to the increased interaction between the lITC and the lIPC in the AD group. Moreover, He et al. found an increased correlation between the supramarginal gyrus and the inferior temporal gyrus in the AD group [18], similar to that found in the current study. The altered connections between the inferior parietal cortex and other brain regions discussed above support the hypothesis that the parietal lobe might be a biomarker for AD [47].

We employed BN models to infer AD/NC group differences by integrating grey matter volume information from multiple brain regions. For the ADNI data, ROC analysis demonstrated that the discrimination had 87.12% specificity and 81.25% sensitivity, and the accuracy rate was 84.53%. Both the sensitivity and specificity were high and were greater than 81%. To evaluate the replicability and stability of our AD-associated BN model, we applied the BN models to an independent dataset acquired using a different scanner. To a certain extent, the data processing step might influence the classification results. To preserve the validity of the classification, the independent dataset must follow the same preprocessing procedures as the dataset used to construct the BN models. For the OASIS data, following preprocessing, the ROC analysis still indicated discriminability with acceptable rates of 83.64% specificity, 80.49% sensitivity and 82.29% accuracy. The accuracy, sensitivity and specificity in the OASIS dataset were high and were similar to those of the ADNI dataset. Although the accuracy of classification for AD vs NC ranged from 70% to 95% [53], [54], [55], most of the studies focused on obvious structural alterations in specific brain regions, such as the hippocampus, when distinguishing the new subjects and did not consider interactions among the different regions. Moreover, there was little trade-off between the indexes of sensitivity and specificity. Our results verified the validity of our AD-associated BN models and supported the hypothesis that the models were universal and scanner-independent. The AD-related BN models can be considered as valid and predictive structural brain biomarker models for AD.

The brain is a complicated network [56], [57], [58] and the DMN is one of the most important resting-state networks affected by AD, which is marked by abnormalities in structural interactions and functional connectivity [23], [26], [27]. The morphological changes in the grey and white matter in different brain regions comply with the covariance pattern, reflecting the network attributes of the human brain. The progress of most neurodegenerative diseases spreads along brain networks, and the network-based hypothesis is supported by researches [59]. We could infer the structural network by analysing the covariance and interactions among different brain regions using structural MRI measurements. Neurodegenerative diseases resulted in abnormal covariant relationships in structure due to grey matter atrophy. A previous study based on cortical thickness indicated that the brain has a “small world” nature and that AD could cause network abnormalities [18]. Furthermore, the structural measurements and the network map could be employed to distinguish AD patients from NC [54], [59].

BN modelling, as a multivariate approach to represent interactions among variables, provided interesting findings in fMRI and has been gradually employed in the study of structural MRI data in recent years. However, BN modelling in structural MRI has mainly detected inter-regional associations that are affected by MCI [20], [21], and the application of BN in the study of AD needs more exploration. In addition, BN analysis of structure was mostly based on discrete variables [20], [21], [60], and there was inevitable information loss during discretization [21]. To overcome this risk, we proposed Gaussian BN models based on continuous variables [43] This approach required the distribution of the continuous variables to be Gaussian; therefore, we verified that the ROI measurements met this requirement in our study. Furthermore, because the distribution of the weight coefficient in each model was unknown, we tested the between-group differences based on weight coefficient metrics using a nonparametric permutation test. The permutation test did not rely on the distribution of the dataset and could sufficiently utilise information from the original sample data, sequentially enhancing the power of the test.

One limitation of this study was that the FLAIR sequences for subjects from the ADNI and OASIS were not available to us to perform direct examination of focal lesions in the white matter. It is necessary to further explore the effect of focal lesions on grey/white matter segmentation in future investigation. Additionally, DTI data were not available for the examination of possible anatomical correspondence of the connections (or co-variations) identified in our study. Finally, we only focused on the DMN, but it would be interesting to investigate abnormality in other resting-state networks that are also affected by AD. Nevertheless, our results demonstrate the feasibility of using BN modelling to further understand the pathological mechanisms of AD, based on the view of the structural network, and of using BN as tool to distinguish AD from NC.
